# Stratapy: a tool for automated stratigraphic log visualisation

**DOI:** 10.1038/s41598-026-58501-2

**Published:** 2026-06-20

**Authors:** Jack Lee Smith, Christina Antoniou, Ruaridh Alexander

**Affiliations:** School of Geosciences, University of Edinburgh, Edinburgh, United Kingdom

**Keywords:** Stratigraphy, Stratigraphic log, Software, Data visualisation, Graphics, Mathematics and computing, Solid Earth sciences

## Abstract

Stratigraphic logs are the fundamental interface between geological observations and scientific interpretation. Manual log visualisation is time-consuming and difficult to reproduce, yet existing digitisation tools are limited and often tailored to specific fields and applications, with many unable to provide core functions. We present stratapy: a Python package for rapid, high-quality log digitisation, designed to be accessible for non-programmers. The tool enables scientists across a range of disciplines to rapidly generate publication-quality stratigraphic logs with basic text- or spreadsheet-based inputs. We have designed stratapy to be accessible even to non-programmers while maintaining a high degree of flexibility in both style and function using a simple parameter-based customisation approach. We incorporate standardised lithological patterns and curated geological features and symbology, as well as automatic correlation with the chronostratigraphic column, the addition of sample locations, annotations and more. For more tailored visualisation, stratapy can easily assemble multi-panel or stratigraphically correlated logs to illustrate complex systems. With applications across research and industry, we create a standardised framework for log illustration and digitisation. This tool will modernise scientific workflows, improving the quality of stratigraphic logs and their interpretation, while contributing towards improved digital practices in the Earth sciences.

## Introduction

Stratigraphic logs are a crucial tool that enable scientists to transform physical, three-dimensional field observations of vertical sections of rocks (lithostratigraphic units) from outcrops or boreholes into graphical representations. Detailed logs also serve as a fundamental tool for the interpretation of observations and the reconstruction of past depositional environments and geological processes and products across various spatial and temporal scales.

Stratigraphic logs are widely used within the geoscience community, and different groups and sub-disciplines have a particular focus, preferring a unique style and format to represent their stratigraphic sequences^1^. For example, sedimentologists are interested in displaying sedimentary structures and products within a stratigraphic sequence to allow for the reconstruction of palaeoenvironmental depositional and/or climatic conditions (e.g., shallow-marine, aeolian, or fluvial systems during wet, humid, or dry climates)^2–5^. Furthermore, the desired level of detail to record sequences is highly variable depending on the research aim. Microscale logs often display high-resolution geological observations, where millimetre to centimetre-scale sedimentary features are important, e.g., laminations or normal grading^6,7^. In contrast, macro-scale sedimentary logs, where several hundreds to thousands of metres of sedimentary units are described, particularly in deep time, are used for section correlation or to identify bulk facies changes over extended intervals^8^. Volcanologists interested in the eruptive history of a particular volcano concentrate on representing unit (e.g., tephra layer) thickness and variable grain compositions (such as lithics and pumice) to illustrate the changing eruptive cycles within a pyroclastic sequence^9–11^. In industry, stratigraphic logs are used to investigate hydrocarbon reservoirs or evaluate ${\text {CO}_2}$ or ${\text {H}_2}$ storage potential, often visualised in composite or correlated logs, or shown alongside complementary datasets^12–14^. Across all disciplines, stratigraphic logs may be standalone or form a minor component of a larger figure e.g., containing palaeontological, sequence stratigraphic, palaeomagnetic, or geochemical data^15–17^.

Variations in visual representations of stratigraphic logs across all disciplines, driven by differences in research objectives, application purposes, or the distinction between academic and industrial use, lead to a need for tailored specifications within a constructed log. The same stratigraphic sequence could be illustrated differently depending on whether it was published in an academic journal or a technical report in industry; it could be produced using two contrasting formats, where symbols, colours, and labels for the same features (i.e., rock lithologies) may vary. Grain size axes may be displayed in high or low resolution, and particular features (e.g., fossils or the presence or absence of minerals) may or may not be displayed for different research purposes despite being present in the stratigraphic sequence. These vast differences in log construction and visual layout affect a researchers’ ability to compare, contrast and interpret logs from similar studies or regions, leading to overly complicated analysis, potential errors in geological modelling, and misinterpretations of geological processes.

Traditionally drawn by hand in the field, logs are often difficult to reproduce digitally, or to share and analyse using modern computational tools. Manual creation and redrafting in design software is time-consuming and a laborious process requiring days or weeks, causing researchers to omit detail, obscure subtle geological features, and reduce visual quality and fidelity of the resulting figure. This reliance on manual practices results in figures poorly suited for integration into digital databases and workflows. As the geoscience community moves toward a “big data” era and aims to leverage the benefits of artificial intelligence, the persistence of non-standardised, poor-quality stratigraphic visualisation represents a barrier to reproducible science, efficient resource management, and improving our collective understanding of the Earth and its processes.

There are a variety of existing tools and software designed to help geologists create stratigraphic logs, all of which have various limitations, with many researchers opting to create their logs manually in design software such as Inkscape, Adobe Illustrator, or others. Table 1 highlights the availability of key logging features in stratapy and other existing software programs. Most available software packages produce tabular outputs for single logs, as opposed to purely graphical representations which are more commonly used in publications and presentations. Some existing tools such as SedLog^18^ and PSICAT^19^ present the user with a graphical user interface (GUI), resulting in time-consuming log creation. With such tools, it can be time-saving to create a simple layout using the software before ‘beautifying’ the tool’s native output in a separate digital illustration software^20^, limiting reproducibility and reducing efficiency and functionality. Other programming-based software such as litholog^20^ and StratiLib^21^ offer more flexibility but are limited in their functionality and customisation capability, as well as sometimes containing insufficient or outdated documentation.

In addition, existing packages contain a limited range of lithological patterns and customisation capabilities, with a default range of only a handful of colours and simple patterns available in many cases. Although some standardised sets of lithologies and features exist, including the United States Geological Survey (USGS) lithological swatch collection, those patterns are generally not integrated into existing tools, with some only providing smaller sets of patterns and features. Furthermore, most existing log generation/visualisation tools do not provide mechanisms for simple functionality. A legend is a critical feature on any stratigraphic log, particularly one that will consider the chronology of lithologies present in the stratigraphic sequence and incorporate all other non-lithological features (e.g., fossils, minerals, sedimentary structures and so on), yet very few existing tools enable automatic legend generation. Customisation of the layout, display or style of the logs (e.g., simple log, grain size axis) is also often not achievable or easy, nor do many existing tools have methods to implement additional features such as complementary chronostratigraphy, trends (such as palaeocurrents or palaeo sea-level indicators), or sample locations.

To address limitations in existing stratigraphic logging software, we present stratapy: an accessible, user-friendly and open source Python package for generating high-quality, publication-ready graphical logs. It enables rapid and fully customisable log creation, while aiming to be easy-to-use for those with no prior Python experience through its user-friendly design and comprehensive documentation and tutorials. Functionality to add scientific features or indicators such as chronostratigraphy, trends, minerals, features, and more are included, as are ways to rapidly and reliably create logs in bulk, or multiple logs/datasets within one figure. Also offered in stratapy is a built-in, curated selection of all USGS lithological patterns, both coloured and black-and-white, amongst other bespoke lithologies and features made specifically for stratapy, to cater to a range of disciplines. By modernising and standardising stratigraphic log workflows, stratapy is scalable from metre-scale sedimentary outcrops to kilometre-scale petrophysical logs, saving users hours of time while improving visual quality and consistency.

## Results

### Software architecture

**Fig. 1:**
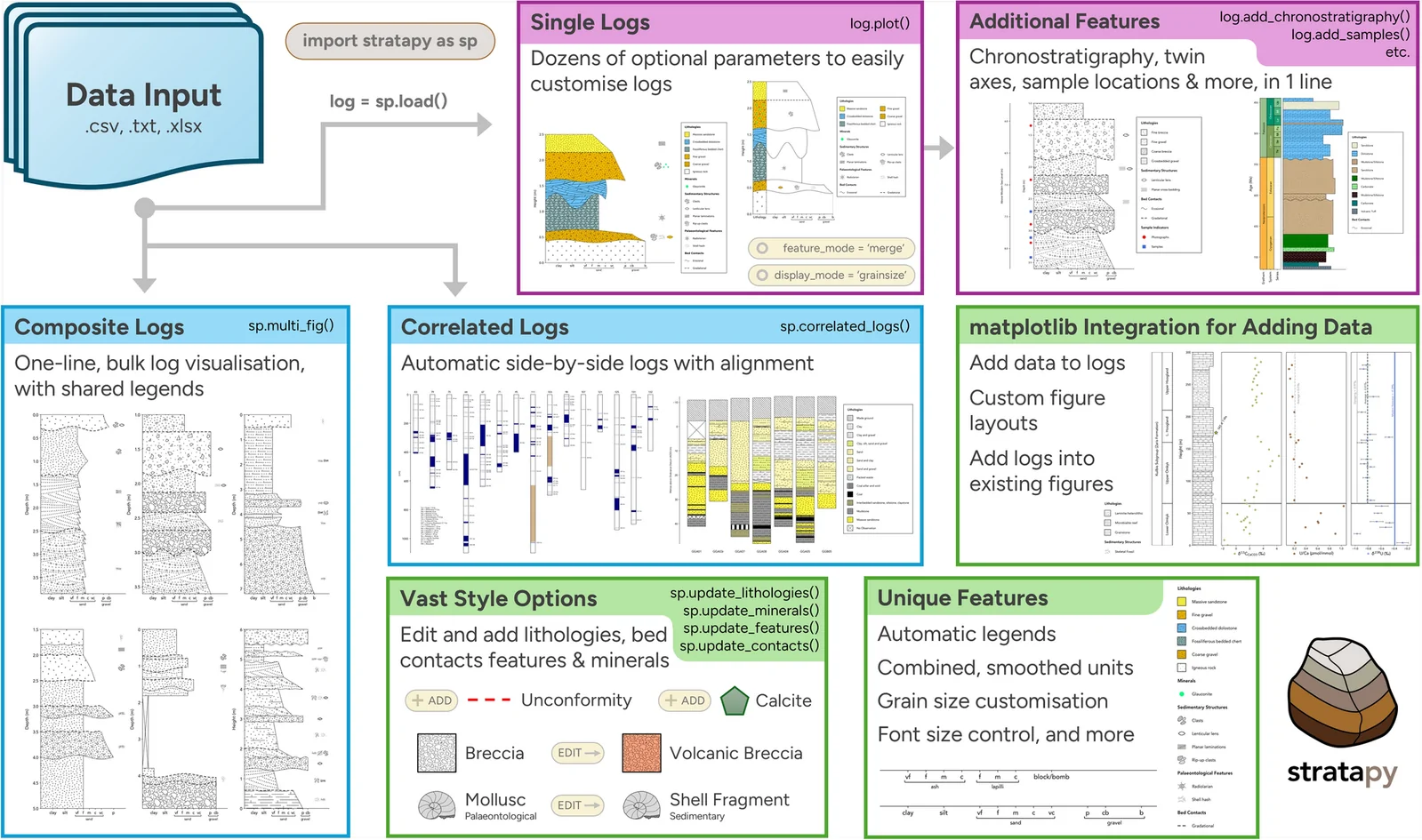
Illustration of the main functions and features of stratapy. Data are taken from literature^2,8,31,45^, as well as the authors’ own research. Each sub-figure in this diagram has been produced directly in Python using stratapy with no subsequent editing; the majority of these examples are discussed in more detail later in this work or are available to view or reproduce in the accompanying documentation and example notebooks.

An overview of stratapy’s architecture is shown in Figure 1. Developing a Python package was favoured over constructing a GUI or software application to enable a wide range of users to exercise their needs, offering varying levels of flexibility, customisation, and control of the resultant logs through an easy-to-use programming language. A code-based approach also ensures that logs can be created in a robust and repeatable manner, with files saved and re-run to reliably recreate the same outputs at a later time, if desired.

**Fig. 2:**
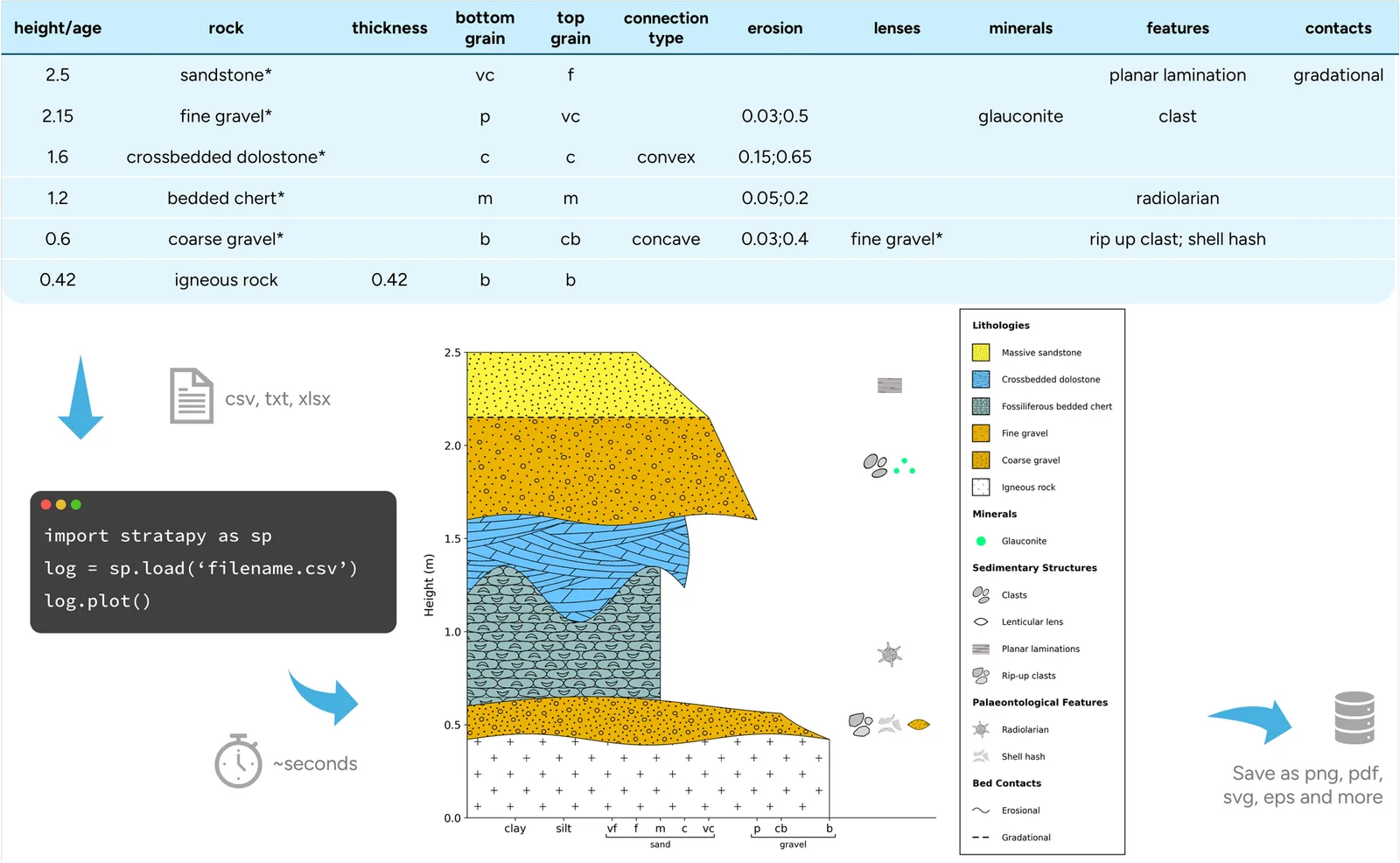
A minimal working example to create a log with stratapy. The tabular input structure is saved as a CSV, TXT, or XLSX file and three lines of Python code are used to create the log as shown, with no subsequent editing.

Users can either download stratapy to their own device, or can use web-based coding environments to run code online, eliminating the need to download and install any program language or software on a device (although stratapy can of course still be used in this way). To create a log, users provide an input file (e.g., .CSV, .XLSX) and can generate a log in seconds from just three lines of code, as illustrated in Figure 2. This design is intended to make it easy to use with minimum effort, even in field-based scenarios where time may be limited.

Logs created with stratapy can easily be placed into existing Matplotlib visualisations^22^, allowing data to be displayed in conjunction with other datasets, as shown in the centre-right panel of Figure 1. This streamlines the creation of complex, publication-quality figures by enabling secondary data to complement stratigraphy, bridging the gap between raw field observations and sophisticated, informative visuals. In addition, given individual preferences between users and disciplines, stratapy’s interoperability enables logs to be exported in formats including, but not limited to, scalable vector graphics (SVG), postscript (PS), or encapsulated postscript (EPS), in addition to other raster or mixed formats like PNG, PDF, JPG, and others.

Digital logs are often not reproducible by others in the community if original design files are not provided. Workflows become complex and prolonged when errors or additions lead to lengthy procedures to edit a static graphic. In stratapy, such issues are negated by enabling users to reproduce figures from an original input file or piece of code. Once generated, stratapy logs can be imported into other graphic design software packages, such as Inkscape or Adobe Illustrator, to allow manual adjustments of the output, if necessary.

Extensive documentation is available online (see ‘Data Availability’) which explains how to install, use, and work with stratapy. It contains examples and tutorials for users of all levels of prior programming experience to easily use. In addition, a gallery of figures provides the input files and code required to reproduce a vast range of logs, which can easily be copied and edited as necessary. All customisation and style options are explained in the documentation, split into short sections for each customisable aspect of the log, but are summarised in the following section.

### Function and capability

#### Usage

Successions in stratapy are built on a bed-by-bed basis, with eleven parameters controlling the customisation of each bed as summarised below, though full details can be found in the online documentation.

- *Height/age* – the position of unit tops; sequences can be represented in height, depth, age, or any other monotonic reference frame
- *Rock* – lithology of this unit from the built-in catalogue or customised patterns
- *Thickness* – thickness of the unit, using any preferred metric system (autofills if left blank)
- *Bottom_grain* and *top_grain* – describes intra-bed grain size variation from a range of presets or customisable values
- *Connection_type* – describes intra-bed grain size transitions (e.g., sawtooth, smoothed)
- *Erosion* – controls the geometry of basal contacts to indicate erosive, undulatory, irregular or complex bedding surfaces
- *Lenses* – indicates presence of intra-bed lenticular beds from the range of available lithological patterns
- *Minerals* – indicates the presence of minerals in the bed
- *Features* – displays palaeontological, sedimentological, volcanological and tectonic features
- *Contact* – defines basal boundary types (e.g., gradational, sharp)

To produce the simplest stratigraphic log, only the lithology and height/age columns are required. Once input files have been created, which could also be done on-site in the field, the simple workflow design of stratapy means logs can be produced quickly starting from three lines of code, aiding ease-of-use for those inexperienced with Python. Figure 2 provides an example input table, alongside the three lines of code required to produce the log. Those new to programming can copy and paste examples from stratapy’s documentation, changing only the name of their desired file. Once input files have been prepared, many logs can be produced automatically.

**Fig. 3:**
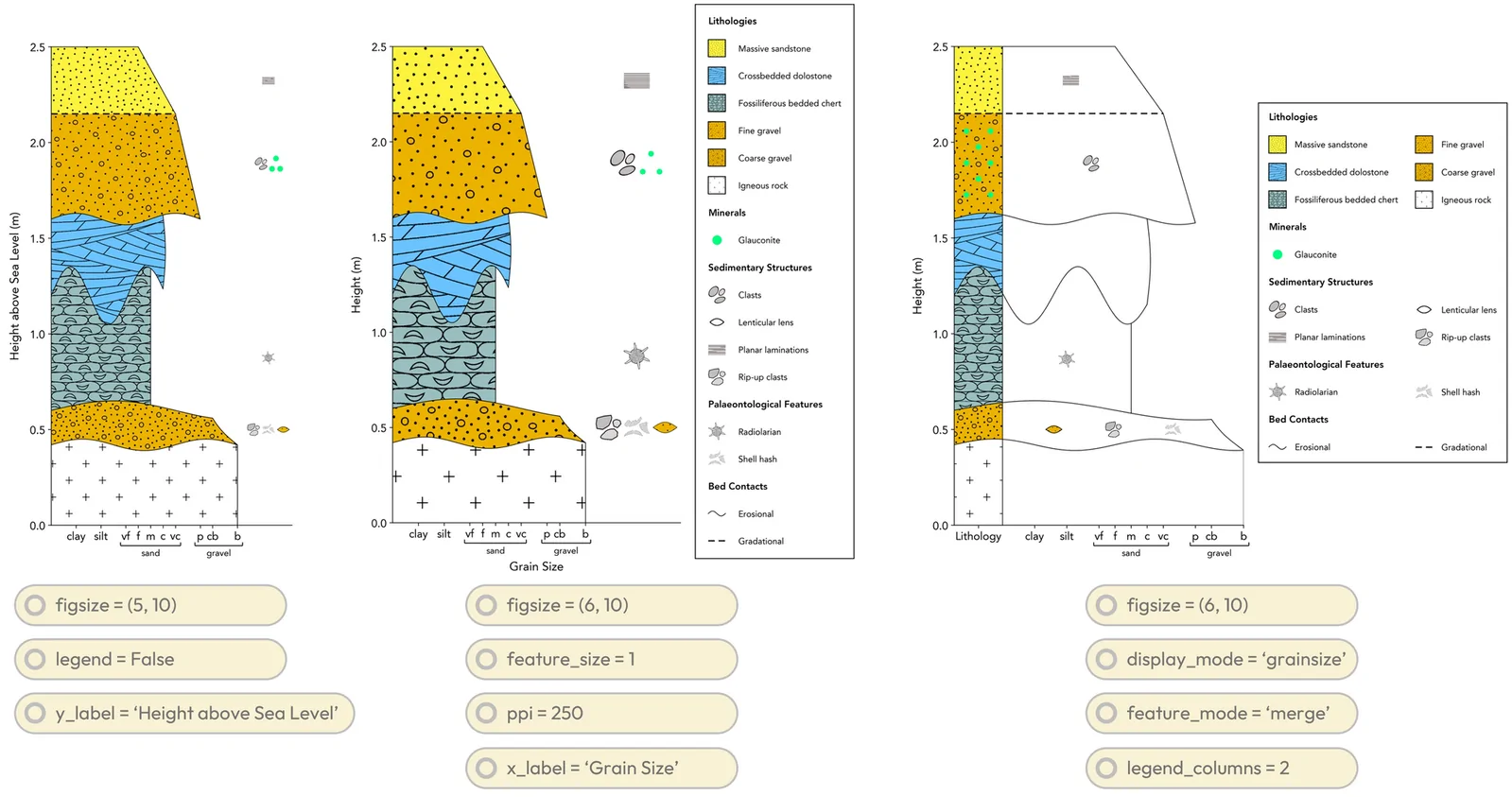
Three logs created using the input file shown in Figure 2 with use of some of the available parameters demonstrated to customise the log format and appearance of the log. Further layout and style configuration options, including the addition of chronostratigraphy, sample markers, and other secondary data, are illustrated throughout the paper and other figures. Each of the three logs are direct outputs from stratapy with no subsequent editing.

A field-drawn sketch-log can be converted to a CSV file in a matter of minutes, which is especially valuable if dealing with numerous stratigraphic sequences or time constraints. Creating digital logs by hand is extremely time-consuming, therefore, stratapy can quickly produce standardised, high-quality logs with enhanced abilities such as, but not limited to, correlated logs to compare stratigraphic sequences across a study area, even with no prior programming experience. Different disciplines require a variety of log types to appropriately display their data. As such, stratapy provides the flexibility required to generate a range of log types. This includes multi-panel logs, simple logs with no grain size component, and logs with grain size variations indicated. These changes to the style or layout of logs are achieved through stratapy’s simple, ‘parameter-based’ customisation approach, as illustrated in Figure 3. Through these design features, stratapy enables easy and intuitive configuration of a log or figure from a single input file.

Ease-of-use has been demonstrated through feedback from a range of researchers who have used stratapy throughout its development. One current user of stratapy notes that 10 logs of 8–10 m thick sedimentary successions, incorporating multiple features and custom lithologies, which would usually take around 3 days to produce using their previous favoured method, can now be produced using stratapy in approximately 30 minutes, with limited coding experience. Log construction time may increase depending on the complexity of the stratigraphic sequence and the volume of data to visualise, however stratapy offers a platform through which the process of construction is simplified and outputs are easily reproducible. In addition, the software is complemented by thorough documentation and its GitHub page, as well as online interactive notebooks which enable users to construct logs without any installation (see ‘Data Availability’).

#### Automatic amenities

Logs produced in stratapy will automatically produce a structured legend to complement one or more logs within a figure. The legend is split into multiple customisable subcategories for improved interpretation, as illustrated in the figures throughout this work. Lithologies used for units and lenses are ordered from the base to the top of a succession. Minerals and features are also integrated into the legend, with the latter split into sedimentary, palaeontological, or tectonic features; erosional, gradational, or sharp bed contacts between units are also summarised in the legend. In addition to controlling the naming and placement of legend elements, the position and layout of the legend can be easily configured through the parameter-based customisation system, or a standalone legend can also be created to accompany more complex figure plates, or to suit individual needs.

Units placed consecutively within a log will be automatically merged into one ‘combined’ unit, while any minerals, features or lenses within those constituent units will continue to be displayed within that stratum only. This powerful feature not only removes unnecessary bed contacts within uniform strata, which most existing software display, it also allows for more precise grain size changes within strata and controlled placement of features/minerals, enabling more representative and scientifically rigorous digital logs through the accurate portrayal of complex units.

In many cases, existing software use a tabular format to visualise grain size within a log, while others use a fixed grain scale, typically from silts and sands through to gravels and cobbles. While useful in some disciplines, other fields would not find use within these constraints. Multiple grain size presets (e.g., lower right panel, Figure 1) are offered in stratapy which change the labels and positions of the grain size markers: clays, silts, sands, and smaller sand sizes for sedimentologists, as well as tephra, lapilli, and blocks/bombs for volcanologists, for example. We also include grain scales based on the extended Dunham classification^23,24^ and that of Leighton and Pendexter^25^ to aid representation of carbonates, as well as a more general sedimentary scale and the ability for users to define their own custom grain size format, if desired.

#### Curated and bespoke symbology

Geoscientists frequently need to overlay specific minerals, fossil assemblages, or tectonic features, and represent localised lenses of alternating lithology to capture the true heterogeneity of a sequence and to highlight features of particular note. However, existing logging tools often fail this requirement, offering limited, non-standardised patterns that lack the flexibility to handle complex successions, or to be customised by the user. The USGS offers a robust set of standardised patterns which can be integrated into some digital tools, but inconsistent support leads to a lack of visual consistency across the community. While existing standards cannot be completely exhaustive, they often neglect particular disciplines like volcanology, leaving researchers to adapt other lithologies, or create logs manually. This lack of a comprehensive, standardised, and extensible digital symbology limits the ability of geologists to create truly precise and universally readable stratigraphic logs.

In addition to making all black-and-white USGS lithologies and patterns^26,27^ available for use by default in stratapy, coloured versions of each lithology have also been provided based on common usage within the community. Furthermore, additional patterns have been curated to build on the USGS collection, adding commonly used patterns like mudstone to the default library. This extensive collection of over 200 patterns should provide sufficient utility for all researchers, however, stratapy also enables customisation of every pattern to allow ultimate flexibility for the user. Each lithological pattern can be adjusted to change the background colour or legend display name, while custom images can also be uploaded for use, if desired. Moreover, single-colour fills are also available to illustrate soils or other unit types. This customisation feature enables any lithology to be represented (whether as a unit or lens) using stratapy. Such a standardised offering is intended to provide a strong data library for the community, and for possible future artificial intelligence or machine learning applications.

The same is true for minerals and features; a bespoke set of more than 75 palaeontological, sedimentary, and tectonic features are built into stratapy, in addition to a range of commonly used minerals. These features cover a wide range of disciplines and research-interests and have been designed with more detail and consistency than many existing collections. These assets can also be configured to individual needs, whether through using custom images, renaming features, or moving them between sections of the legend. Minerals can similarly be added as required, or existing mineral styles can be edited in colour and shape, as illustrated in the lower middle panel of Figure 1. These vast customisation options ensure that all users, regardless of discipline, research interests, or field-specific constraints, will be able to accurately and professionally represent their observations with a common palette of lithologies, minerals, and features.

## Discussion

The previous sections have outlined the architectural principles and technical goals of stratapy. The following use cases demonstrate its practical utility using published datasets from the fields of sedimentology, volcanology, and palaeontology and geochemistry, as well as industrial applications. The remaining figures in this paper are direct outputs from Python using stratapy, with no subsequent editing.

### Sedimentology

**Fig. 4:**
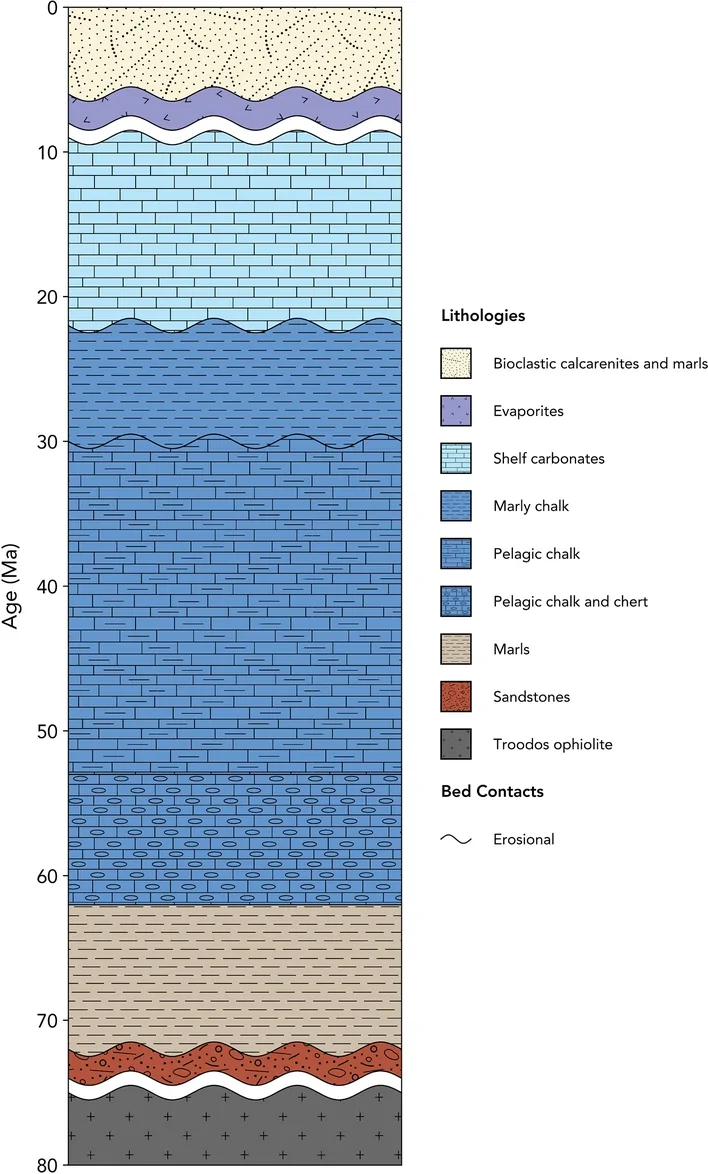
A stratigraphic log illustrating the depositional history of southern Cyprus, created with a customised style and automated legend. Recreated from^46^ using stratapy with no additional editing.

Figure 4 presents a composite stratigraphic column summarising the key geological units from the circum-sedimentary cover of the Troodos ophiolite in southern Cyprus (Eastern Mediterranean), recording a succession from the Upper Cretaceous to the Quaternary. It demonstrates key functionalities and the basic principles of log construction using stratapy, including customising lithologies, excluding the grain axes by implementing the ‘log’ layout setting, and the relabelling of units for the legend.

**Fig. 5:**
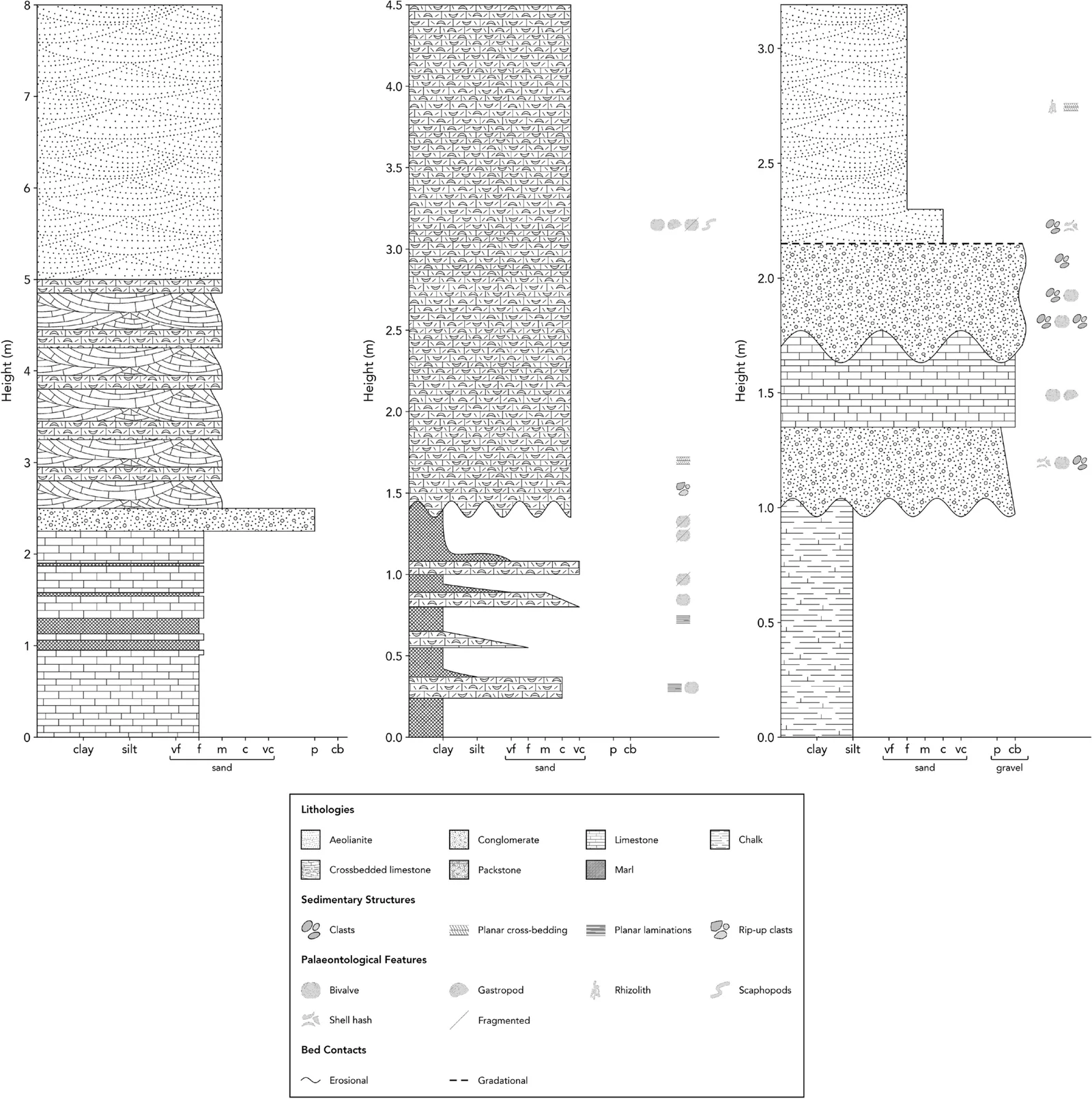
Sedimentary logs plotted side-by-side illustrating sequences from the Messinian (left), Pliocene (middle), and Pleistocene (right), as shown in Figure 4. Figures recreated from^2,28,29^ using stratapy with no additional editing.

Figure 5 complements the composite log using three sedimentary logs, presented side-by-side, illustrating sedimentary units within the Messinian, Pliocene, and Pleistocene deposits of southern Cyprus extracted from the section in Figure 4. The detailed sedimentary logs in this figure are constructed from additional sources to feature some of the basic capabilities of stratapy^2,28,29^. This figure is created using a single line of code in stratapy, which plots each log on its own height axis, with harmonised symbology and a shared legend enabling direct comparison of lithologies, bed thickness, and key contacts while preserving local scale. As demonstrated, stratapy’s single-line multi-figure functionality is used to plot many files in a 1D or 2D grid with a complementary legend and optional alignment of the abscissa and ordinate axes. Additional customisation through renaming existing lithologies and features, as described in previous sections, have also been used to create these logs.

Where additional cartographic elements or bespoke annotations are desired, stratapy figures can be extended (e.g., adding text labels with Matplotlib) or exported to layered SVG/PDF files for light vector editing. Additional data-driven elements like chronostratigraphy, sample markers, or a twin y-axis (see Figure 1), can each be added with stratapy with one additional line of code. With sedimentary logs often shown in groups as collected from field localities, Figure 5 illustrates the advantages of automating the construction of such figures which can drastically reduce the amount of time in visualising one’s observations.

### Palaeontology and geochemistry

Logs may also be presented alongside other datasets in a composite figure, such as geochemical and fossil occurrence data, or chronostratigraphy and rock formations. Palaeontologists and geochemists studying deep time typically make use of such figures, logging broad lithological changes and correlating them with sea level and bulk lithofacies changes, and presenting multiple datasets. Using stratapy, users have the ability to produce a log that can be customised by automatically integrating chronostratigraphy, as well as a range of sedimentological and palaeontological features, or complemented with multiple axes of auxiliary data. Other frequently used features by researchers in this field are available, such as trend glyphs to denote temporal trends (e.g., coarsening-/fining-upward or shallowing-/deepening-upward).

**Fig. 6:**
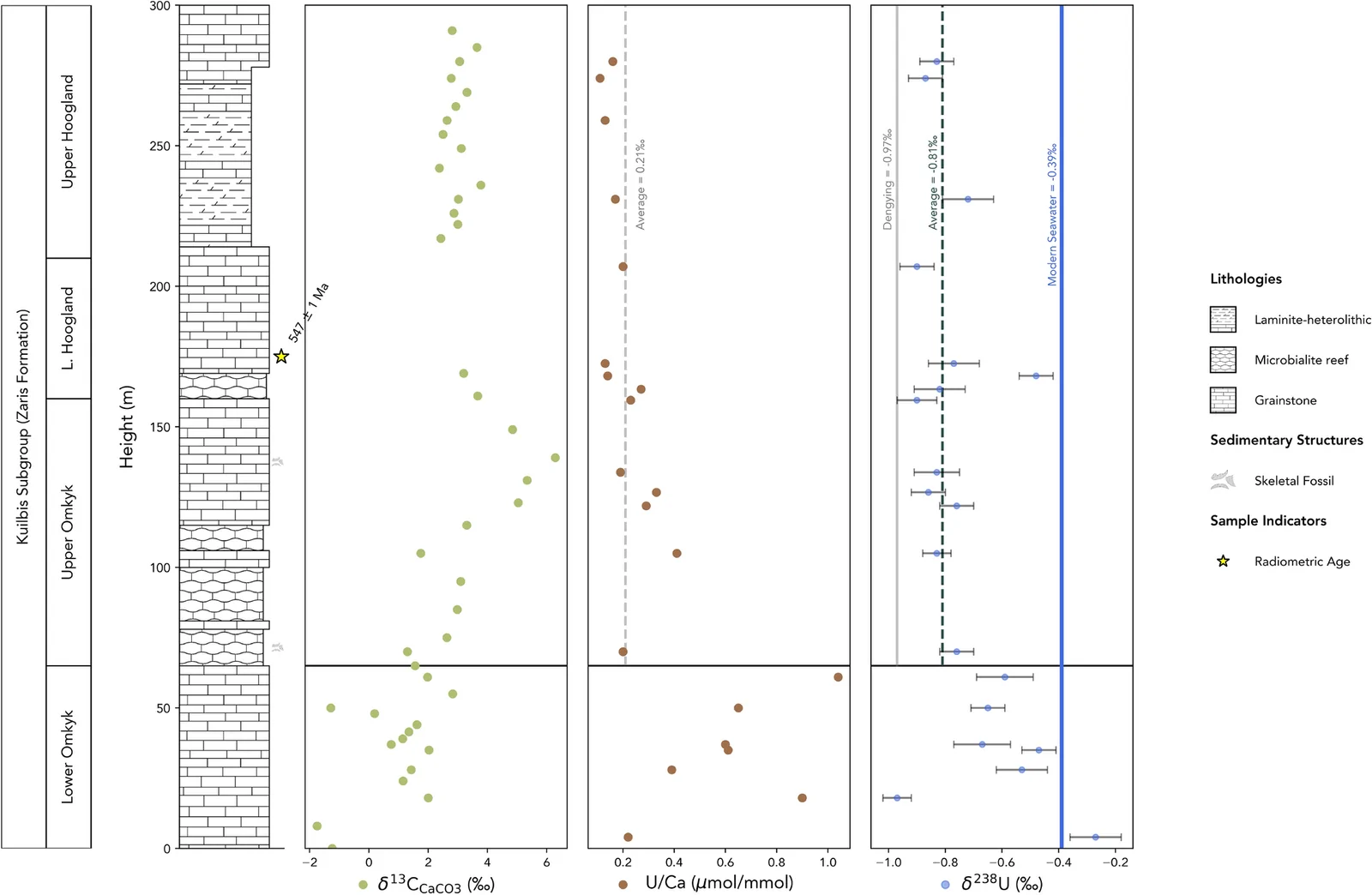
Stratigraphic log with accompanying geochemical data for carbonate rocks from the Kuibis Subgroup of the Nama Group. Recreated from^30^ using stratapy and Matplotlib with no additional editing.

The example provided in Figure 6 shows a sedimentary succession through the late Neoproterozoic of Namibia^30^. The replicated log is produced with three lines of code, with additional features, including fossil occurrences and a radioisotope geochronology date, also added through stratapy’s customisation options. The log is then integrated with four additional data panels for regional formation subdivisions and geochemical data using Matplotlib (Figure 6).

### Volcanology & tephra

In tephrochronology, logs describe tephra layers, sometimes labelled with facies information, radioisotope dating results, thicknesses, and other characteristics^31^. In other cases, the location of samples, photographs, or other features are indicated in a sequence combined with other lithological observations^32^.

**Fig. 7:**
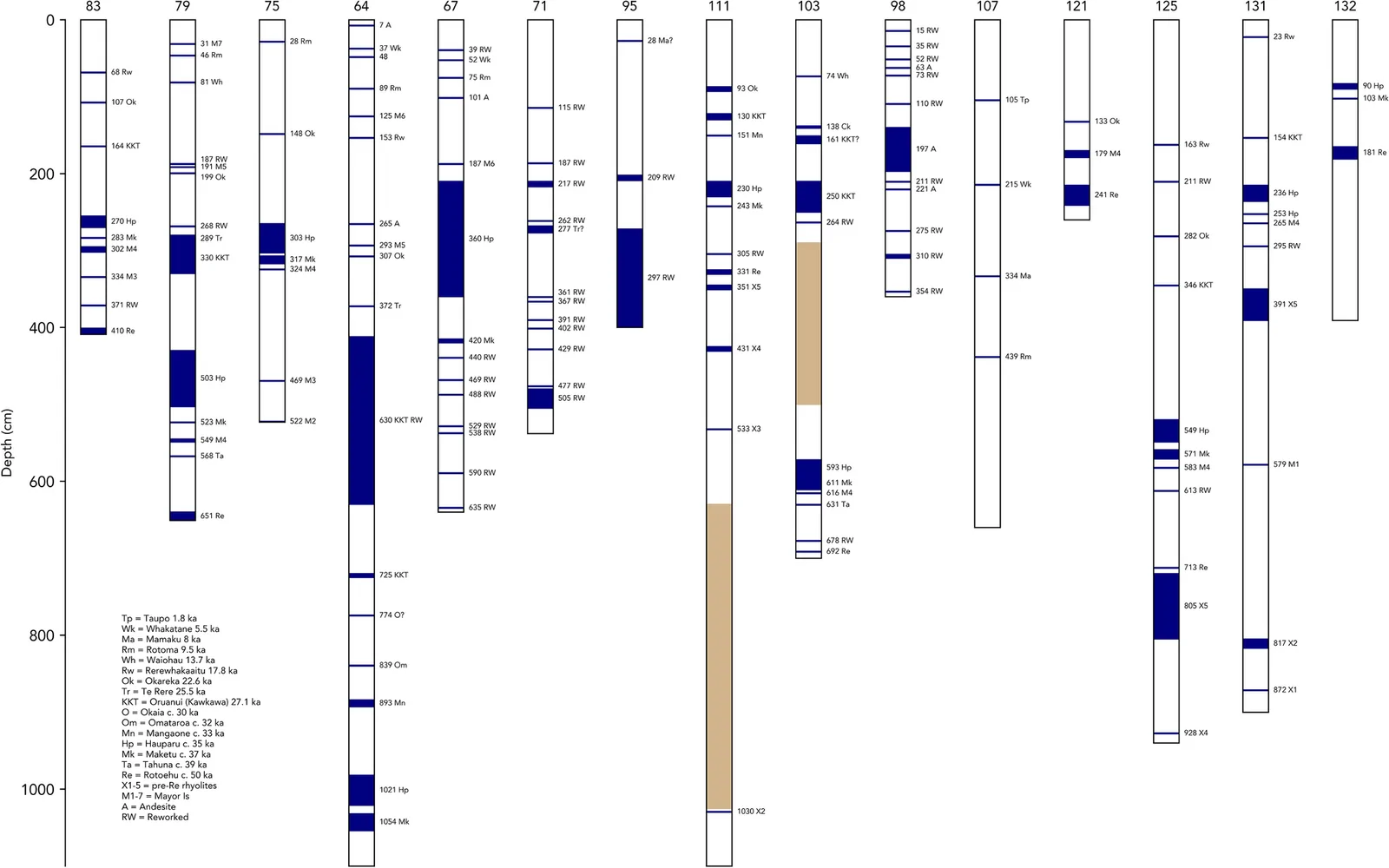
Aligned stratigraphic columns of cores indicating the presence (navy) of tephra layers, as well as regions of sediment separation in the coring barrel (beige). Tephra layers are annotated with their depth at the layer base and their identification. Figure produced by stratapy, recreating Figure 2 from^31^ with no additional editing.

Using stratapy, we are able to display aligned, side-by-side correlated logs to allow for stratigraphic or temporal correlation, enabling users to automatically visualise logs from numerous files with ease. Figure 7 uses correlated logs to illustrate the preservation of large magnitude tephra falls offshore the Bay of Plenty, North Island, New Zealand. This figure indicates the presence and thickness of tephra layers, without any lithological patterns or unit borders, however, custom volcanological patterns are also available by default, if desired. In this case, stratapy has also been configured to produce the figure without a legend to enable manual reference to relevant features, with a key for tephra units created instead.

### Applied geoscience: energy, resources, and infrastructure

**Fig. 8:**
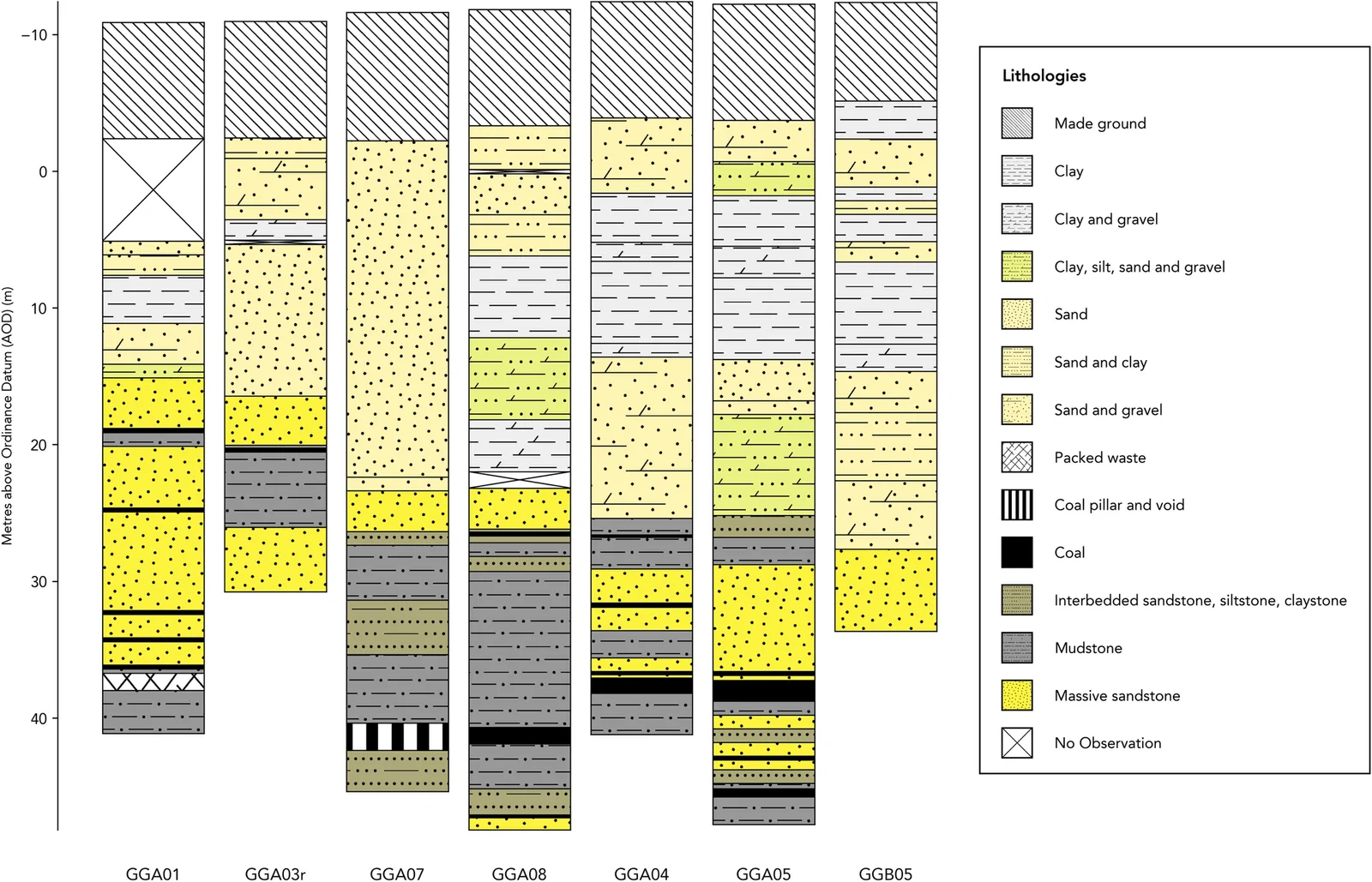
Seven vertically aligned borehole logs (approx. 45 m) from the UKGEOS project’s Glasgow observatory^45^ produced by stratapy with no additional editing.

In industry, correlated borehole data may be displayed in a similar way to the above volcanological datasets. Figure 8 represents a stratapy-reproduced figure from the British Geological Survey’s UK Geoenergy Observatories (UKGEOS) project which assesses mine-water thermal energy storage^33,34^. In this case, several borehole datasets are presented side-by-side in a single figure. The correlated logs are produced with just one line of code, which automatically aligns the boreholes, compiles a legend, and provides a single, shared axis. Additionally, logs can be manually offset to account for subject- or site-specific requirements.

These borehole array data demonstrate that understanding complex processes below ground - whether thermal, or otherwise - requires a unified visual synthesis of the subsurface. By correlating stratigraphic markers across multiple research compounds, engineers and researchers can delineate the continuity of geological units and validate numerical models.

### Educational value

Undergraduate students are commonly taught to hand-draw stratigraphic logs either by recording observations in the field, or completing handouts in a classroom, but the subsequent digitisation of these records is frequently overlooked. Our package offers a new and interactive approach to introducing students to lithostratigraphy and sedimentology, as well as chronostratigraphy, biostratigraphy, and geochemistry.

As modern teaching is increasingly digitised^35,36^, including the uptake of programming languages like Python within geoscience curricula, stratapy offers a simple and intuitive framework through which students can construct their own stratigraphic logs computationally. Rather than treating logs as static, hand-drawn outputs, students can experiment dynamically: adjusting lithological parameters, modifying scale and thickness, exploring symbol conventions, and observing how these changes influence the visual and interpretative structure of a log. At the same time, the transition from analogue field notebooks to structured, digital data formats is a crucial skill in modern industry, helping students develop skills in computational thinking, reproducibility, and integrated geological analysis.

### Conclusion

We have created stratapy to be a free and open source, easy-to-use Python package for the digitisation of stratigraphic logs, which has applications in all fields of the Earth sciences. Its simple functionality, which requires only a few lines of code, makes it an ideal tool for those wanting to construct figures with limited coding experience and/or time. Our software can create single logs, multi-log figures, and integrates into the wider Python ecosystem for ultimate flexibility for the user. The highly customisable nature of stratapy, from the hundreds of available lithologies and features, to automatic utilities like legend generation, automatic chronostratigraphy, and adding secondary data, makes it a more comprehensive and flexible open source alternative to existing tools, as illustrated in Table 1. Although stratapy may not be able to appeal to those who use highly non-standard logging conventions, we have demonstrated that this tool offers a wider range of functionality than existing tools. Customisation of logs to meet a wide range of disciplines and research aims through a simple parameter-based mechanism ensures that stratapy is accessible to all, while producing professional, high-quality figures for those in academia, industry, and the wider community.

As the Earth sciences becomes more digitally-literate, the use of a coding language such as Python for the analysis and presentation of data is becoming more routine and beneficial. Indeed, as universities add digital/computational skills courses to the curriculum, stratapy has the potential to not only be a valuable professional tool, but also a valuable teaching tool. It is hoped that stratapy will promote reproducibility within the logging and geoscience community, as well as encourage consistency between academia, industry and the various disciplines within. The package’s built-in collections of lithological patterns, features and minerals, in addition to its vast customisation options enables data from all areas of the Earth sciences to benefit and find use in this standardised tool.

**Table 1:** Comparison of key features between existing stratigraphic log tools and stratapy.

Stratigraphic log tools					
Criteria	SedLog	StratiLib	litholog	PSICAT	stratapy
User Focus	Sedimentology	Sedimentology	Sedimentology & quantitative analysis	Geological boreholes & cores	All Earth Science disciplines
User Interface	GUI	Code	Code	GUI	Code
Last Updated	2015	2022	2022	2026	2026
Log Format	Tabular log	Tabular log	Graphical log, fixed layout	Graphical log, fixed layout	Graphical log, customisable layout
Log Customisation	✓ Manual selection	✘	✘	✘	✓ Parameter-based customisation
Automatic Legend Generation	✘	✘	✘	✓	✓
Available Lithologies	*≤ 35*	35	4	USGS	*≥ 200* USGS & more
Features	✓	✓	✘	✓	✓
Minerals	✘	✘	✘	✘	✓
Bed Contacts	✘	✘	✘	✘	✓
Asset Customisation	✓ Manual.svg import	✘	✓ Lithologies only	✓ Lithologies & features only	✓ All assets
Built-in Chrono- stratigraphy	✘	✘	✘	✘	✓
Multi-log Figures	✘	✘	✘	✘	✓
Software Integration	✘	SedLog & Python Ecosystem	Python Ecosystem	✘	Python Ecosystem
User Aids	YouTube tutorial, limited documentation	Example notebooks	Limited documentation	YouTube tutorials, user manual	Extensive documentation, tutorials and example notebooks
Export Formats	.pdf, .svg, .jpeg	.png	.png, .jpeg	.pdf, .svg, .png	.pdf, .svg, .png, .jpeg, and any other format depending on your environment

## Methods

Stratapy was built in Python, widely considered one of the most general-purpose, high-level, and easiest-to-use programming languages, combined with an array of existing packages^22,37–41^. The software ingests data of tabular format with a range of geological parameters and constructs a stratigraphic log on a bed-by-bed basis. Input files (CSV/Excel/JSON) are read with pandas and normalised to a canonical DataFrame with one row per bed. The parser validates column data and types, suggesting possible solutions for incorrectly formatted inputs, enforces monotonic sequencing, and standardises categorical values. Each log is represented by a LogObject class which constructs an ordered collection of bed records and orchestrates a rendering pipeline: bed boundaries and polygons are computed in data coordinates; lithological fills are applied via a pattern registry; optional mineralogy and bed-scale features are drawn, followed by contacts and outlines. Lithological patterns were adapted from Daven Quinn’s modern formatting of the Federal Geographic Data Committee (FGDC) Digital Cartographic Standard for Geologic Map Symbolisation^26,27^, while sedimentary, palaeontological, and tectonic features were created by the authors. All are stored and loaded as PNG images. Legends are generated automatically by collecting unique pattern/feature keys from the plotted artists and passing de-duplicated handles and labels to matplotlib’s legend constructor while accounting for ordering, labels, and visibility in line with user preferences. For correlated or multi-panel figures, a MultiLogObject class builds each log independently and then links them by sharing a common axis where desired and unifying scales, offsets, and legends via matplotlib and custom utilities. All plotting functions accept an existing matplotlib axis and return the Axes and artist handles to facilitate downstream customisation.

The package includes a full suite of unit and integration tests implemented using pytest^42^ which cover basic functionality, input-validation checks and ecosystem compatibility. Compatibility across Python versions and dependency ecosystems was systematically validated using nox^43^, which executed the test suite against multiple Python releases as well as current and previous versions of key dependencies. A full compatibility matrix is available in the GitHub README. Continuous integration via GitHub Actions ensures that all tests are automatically executed on every code change.

## Data Availability

stratapy is released under the 3-Clause BSD Licence and can be installed using the pip package installer: pip install stratapy, or accessed through GitHub at https://github.com/Jack6228/stratapy. The latest version of stratapy can always be found at the DOI in reference ^44^. Full documentation, including installation, tutorials, and a gallery of examples, can be found at https://stratapy.readthedocs.io/en/latest/, or through the GitHub and Zenodo links above. A Python notebook to recreate the figures in this paper can also be found in the above GitHub repository, but alternatively can be accessed and run immediately online, without any installation, using this Google Colab notebook: https://colab.research.google.com/github/Jack6228/stratapy/blob/master/examples/ManuscriptFigures.ipynb. An additional tutorial notebook can also be accessed: https://colab.research.google.com/github/Jack6228/stratapy/blob/master/examples/Tutorial.ipynb.
